# St. Mary's Hospital Festival Dinner

**Published:** 1894-06-09

**Authors:** 


### HOSPITAL FESTIVALS AND MEETINGS.

### ST. MARY'S HOSPITAL FESTIVAL DINNER.

The presence of the Duke of York in the chair on the occasion of the annual festival dinner of St. Mary's Hospital last Friday at the Hotel Metropole was, of course, perfectly natural. His Royal Highness not only is, and has been since 1892, president of the institution, but he and his family have thoroughly identified themselves with the erection of the new Clarence Memorial wing. This extension of work is, of course, all paramount in point of interest with those who follow the fortunes of the Paddington Hospital, but singularly enough very little reference was made to it the other night except by Sir W. Broadbent. Unfortunately, however, it happens to be the case that the existing work of the hospital is just now in want of support, for there was a very serious falling off in its income last year, and over £7,000 had to be drawn from invested funds. This falling off was almost entirely on legacies, which only amounted to £3,047 against £10,557 in the preceding year, a fact which once more emphasises the undesirability—to use no stronger term—of treating these receipts as current income, and which, to a very great extent, corroborates the opinion entertained in philanthropic and charitable circles to the effect that testamentary bequests of this character are not numerically on the increase. The chairman of the Board of Management showed, too, that he was alive to the fact that the new death duties will probably exercise a marked effect on legacy-leaving, but if this takes the form of increased liberality during the lives of the donors there will be no cause for regret at a healthier state of things. The deficit to which allusion has been made necessitated an appeal on behalf of the general maintenance fund conjointly with the Clarence Memorial wing, which was the sole object of the appeal made at the dinners in 1892 and 1893, in the former case with almost phenomenal in the latter with rather disappointing results. Last Friday's subscription list can hardly be regarded as anything but satisfactory. Though by no means the largest sum collected as yet this year at philanthropic festivals, £5,282 10s. is a substantial amount, and was not accounted for by any large individual donations, the Duke of York, by the way, contributing twenty-five guineas. Some 150 guests attended the dinner, including Cardinal Vaughan, the Marquis of Bath, Sir Charles Hall, M.P., Admiral Sir A. Phillimore, Admiral Sir W. H. Stewart, Admiral Sir Erasmus Ommanney, Major-General Sir F. W. de Winton, Colonel Stanley G. Bird, Colonel Lewis V. Loyd, M.P., General Lowry, Sir W. Broadbent, the Hon. H. L. Noel, General Moberly, Colonel-Surgeon A. T. Norton, Colonel Blair, Colonel Birch, Colonel W. B. Hamilton, Colonel Bannerman, Mr. J. Aird, M.P., Mr. F. C. Frye, M.P., Mr. M. H. Shaw-Stewart, M.P., Mr. E. H. Hulse, M.P., Mr. W. Holland, M.P., Mr. Alfred de Rothschild, Dr. Braxton Hicks, Dr. Lee, Dr. Symes Thompson, D. Mitchell Bird, Dr. Scanes Spicer, Dr. Hammond Smith, Dr. Sidney Phillips, Mr. Leonard Bonn, Mr. Malcolm Morris, Alderman Beachcroft, L.C.C., Mr. G. P. Field, Mr. J. R. Mellor, Mr. H. W. Page, Mr. H. J. Alcroft, Mr. H. Oppenheimer, and Mr. M. P. Christie.

The Chairman having, according to wont, very briefly proposed the toast of "The Queen," Cardinal Vaughan followed with that of "The Princess of Wales and the Other members of the Royal Family," and in the course of a few felicitious remarks observed, amid much laughter and cheers, that though we were a fairly discontented people, loving to find fault with ourselves and our own institutions no one ever heard anything like an adverse comment on the Sovereign or the Royal Family. In acknowledging the warm reception which the toast received, the Chairman pointed out that his father had, on two occasions, laid the foundation stones of additions to St. Mary's Hospital, in 1868 and in 1892—(cheers)—and that his aunt, the Princess Louise, had opened a new wing in 1884, thus showing that his family had taken a practical interest in the hospital for many years. (Cheers.) He was proud to occupy the position of president of St. Mary's Hospital, which did so much to alleviate pain and suffering. Pausing thence to propose the toast of "The Navy, Army, and Reserve Forces," his Royal Highness laid especial stress on the fact that that day was the centenary anniversary of Lord Howe's great victory over the French, an event which formed, too, the theme of Admiral Sir E. Ommanney's remarks who responded on behalf of the Navy. General Lowry once more replied for the Army and Colonel-Surgeon Norton for the Reserve Forces.

The Chairman then said: I have now the pleasure of proposing the toast of the evening, which is "Success and Prosperity to St. Mary's Hospital"—(cheers)—and I feel sure you will give it a hearty reception. (Cheers.) Fifty-two years ago St Mary's Hospital was founded to meet the wants of Paddington, West Marylebone, and North Kensington. The foundation-stone was laid in 1845 by my grandfather, the Prince Consort—(cheers)—and since then the hospital has ministered to the wants of many thousands of our suffering poor. With regard to its administration I should like to mention the following facts, which I think speak for themselves. Bed for bed, St. Mary's Hospital does more work than any general hospital in London having a medical school attached to it, and the average cost per bed is under that of any of the large institutions of a similar nature. (Cheers.) But we are met with this difficulty: our hospitals were not able to keep pace with the rapid growth of London, and the enormous increase of her population. Each in its turn has had to be enlarged, St. Mary's among the number, and we feel we can no longer delay bringing our wants to the public notice and asking for its kind support. As president of this hospital—(cheers)—I feel some diffidence in making this appeal lest my connection with it might lead to the supposition that I am biassed in its favour—("No, no")—and I also wish that some more eloquent chairman than myself had presided this evening—("No, no")—who could have pleaded with you on its behalf with greater eloquence than I possess, though not with greater earnestness than I do. (Cheers.) Briefly, then, we want £7,000 to replace the sum which had to be withdrawn from our funds to meet the deficit incurred last year with our working expenses, and we also want £12,000 to complete the sum necessary to build the new out-patient department, which is so sorely needed, and which forms part of the new Clarence Memorial wing. (Cheers.) These are our wants, and while we acknowledge with great thankfulness the warm and hearty support we have ever received in the past, we ask you and all who live in the neighbourhood of Paddington, whose suffering poor find relief within our walls, to help us in our hour of need, in order that we may help those who are unable to help themselves—the sick and poor. (Cheers.)

Colonel Stanley Bird, the chairman of the Board of Management, was very brief in his reply, chiefly devoting himself to a eulogy of the help the Royal Family had rendered to the hospital, such assistance being never more wanted than at the present time. He considered that it would be a suicidal policy to close any of the wards, and while he felt that they owed a debt to the public, he thought the public owed a debt to them, and ought, by coming forward, to pay it, to assist them a great deal more than they did. Still, he did hope that better times were in store for the hospital.

Sir Charles Hall, in an anecdotal vein, proposed "The Medical Staff," to which toast Dr. Broadbent responded, with a very earnest appeal on behalf of the much-needed proposed extension of the hospital, and a warm testimony to the deep interest which the medical staff of the institution take in its progress. With this appropriate reply the proceedings terminated, but the secretary, Mr. Thomas Ryan, to whose energy this great success is mainly due, had previously been able to announce the subscription list of over five thousand guineas.

